# Willingness of using rapid diagnostic tests for malaria and implications of concurrent availability of HIV tests in central Côte d’lvoire

**DOI:** 10.1186/1475-2875-11-S1-P73

**Published:** 2012-10-15

**Authors:** Colombe Coffie Comoé, Allassane F Ouattara, Giovanna Raso, Marcel Tanner, Jürg Utzinger, Benjamin G Koudou

**Affiliations:** 1 Département Environnement et Santé, Centre Suisse de Recherches Scientifiques en Côte d’lvoire, Abidjan, Côte d’lvoire; 2 Département de Sociologie, Université de Cocody, Abidjan, Côte d’lvoire; 3 Laboratoire de Cytologie et de Biologie Animales, UFR Sciences de la Nature, Université d’Abobo-Adjamé, Abidjan, Côte d’lvoire; 4 Department of Epidemiology and Public Health, Swiss Tropical and Public Health Institute, Basel, Switzerland; 5 University of Basel, Basel, Switzerland; 6 Vector Group, Liverpool School of Tropical Medicine, Liverpool, UK

## Background

Mortality due to malaria is largely driven by inadequate and/or delayed diagnosis and case management. The development and effective use of rapid diagnostic tests (RDTs) have contributed to reduce malaria mortality and morbidity, and hence RDTs have become essential tools for the control and elimination of malaria, particularly in areas where health services are deficient. We determined the knowledge, attitudes, practices and beliefs in relation to RDTs in two communities of central Côte d’lvoire.

## Methods

One hundred suspected malaria cases from Bozi and Yoho visiting the health centre in Bozi, were interviewed in April 2010 by administering a pre-tested questionnaire on current practice and perceptions of RDTs. The relationships between acceptance of RDTs and factors related to opinions were identified, using generalized linear mixed models. Qualitative data from open-end responses complemented the quantitative analysis.

## Results

More than half of the people interviewed (54%) perceived blood as a *“sacred body fluid”*, while less than half complied with RDTs (44%). The concurrent availability and use of RDTs for HIV testing at the same health facility was associated with an unfavourable attitude towards RDTs for malaria (Fisher’s exact test, p <0.001). The initial willingness of patients to accept malaria testing with RDTs was significantly related to general fear and wanting to know malaria infection status. For further and regular use of RDTs, an association was observed between the acceptance of RDTs and the wish to be tested for HIV (odds ratio (OR) = 16.6, 95% confidence interval (CI) = 1.03-236.5). Those thinking that blood samples were useful for medical diagnoses were 8.3-times (95% CI = 2.2-31.1) more likely to undergo an RDTs compared to those rejecting blood sampling as a diagnostic strategy.

## Conclusions

Socio-cultural factors might be barriers for accepting RDTs in general health services. There are social representations of malaria and HIV/AIDS, symbolic for blood or experiences in relation to blood sampling and blood-related diseases that might challenge the introduction and routine use of RDTs. These barriers must be given special attention to further promote RDTs for prompt and effective diagnosis and adequate management of malaria.

